# Sensory Modulation Disorder as a Diagnostic Marker in Fibromyalgia: Associations with Stress and Symptom Severity

**DOI:** 10.3390/diagnostics15212700

**Published:** 2025-10-24

**Authors:** Patricija Goubar, Tomaž Velnar

**Affiliations:** 1 Faculty of Health Sciences, Alma Mater Europaea University, 2000 Maribor, Slovenia; tvelnar@hotmail.com; 2 Department of Neurosurgery, University Medical Centre Ljubljana, 1000 Ljubljana, Slovenia

**Keywords:** fibromyalgia, sensation disorders, biomarkers/diagnosis, discriminant analysis, psychological stress, chronic pain, questionnaires, central nervous system, diagnosis, differential, diagnostic markers

## Abstract

**Background/Objectives**: Fibromyalgia (FM) is a nociplastic pain disorder marked by altered central nervous system processing and abnormal sensory modulation. Diagnosis remains largely symptom-based and lacks objective biomarkers. Sensory modulation disorder (SMD)—impaired regulation of responses to non-noxious input—may represent a clinically relevant diagnostic dimension. This study aimed to estimate the prevalence/diagnostic value of SMD in FM, examine links with symptom severity and stress, and assess its potential for patient stratification. **Methods**: In this cross-sectional study, 182 adults were enrolled (104 FM; 78 controls). Standardized instruments included the Adolescent/Adult Sensory Profile (AASP), Fibromyalgia Impact Questionnaire (FIQ), and Perceived Stress Scale (PSS). Group comparisons, regression, and discriminant analyses evaluated SMD profiles. **Results**: Compared with controls, FM adults showed higher sensory sensitivity and avoidance (both *p* < 0.001), lower sensation seeking (*p* = 0.002), and modestly higher low registration (*p* = 0.027). Elevated SMD correlated with greater symptom severity and perceived stress. Stress significantly predicted FM’s impact (β = 0.57, *p* < 0.001). A discriminant model achieved 84% apparent in-sample accuracy for classifying FM severity from sensory/stress profiles. **Conclusions**: Sensory modulation abnormalities are highly prevalent in FM and show meaningful associations with symptom severity and stress, suggesting that SMD could represent a potential diagnostic dimension and stratification aid. These findings should be interpreted within an exploratory, cross-sectional design. Incorporating sensory modulation assessment into FM evaluation may improve diagnostic precision, reduce delays, and guide individualized management. Confirmation in larger longitudinal studies is warranted.

## 1. Introduction

Fibromyalgia (FM) is increasingly recognized as a prototypical nociplastic pain disorder, defined by altered central nervous system (CNS) processing and impaired pain modulation rather than by peripheral tissue damage or inflammation [1]. Current estimates suggest that FM affects approximately 2–4% of the global population, with women disproportionately affected, leading to a considerable personal, social, and economic burden [2,3,4]. Beyond direct disability, FM imposes high health-care costs, reduced work productivity, and diminished quality of life, highlighting its significance as a public health challenge [5].

The clinical picture of FM is notably heterogeneous. Core features include widespread musculoskeletal pain, persistent fatigue, non-restorative sleep, cognitive dysfunction, and affective disturbances [6]. However, this symptom constellation is rarely static. Patients often describe fluctuations in symptom intensity over time, often triggered by stress, physical exertion, or environmental factors. This variability complicates diagnosis and management. Moreover, FM symptoms overlap extensively with other chronic pain and stress-related conditions—such as chronic fatigue syndrome, irritable bowel syndrome, rheumatoid arthritis, and mood disorders [7]. These overlaps create a diagnostic gray zone in which FM may be dismissed as psychosomatic or incorrectly attributed to other rheumatologic or psychiatric disorders. Such misclassification not only hinders timely treatment but also contributes to stigma and patient frustration. Consequently, a key clinical priority is the identification of more objective and reproducible diagnostic markers that can reliably distinguish FM from related syndromes [8].

Emerging evidence strongly implicates CNS dysfunction as the principal driver of FM pathophysiology. Central sensitization, impaired descending inhibitory control, and altered sensory gain have been consistently observed [9]. Advanced neuroimaging studies over the past decade have suggested structural and functional alterations in brain regions central to sensory and affective integration, such as the insula, anterior cingulate cortex, and prefrontal cortices [10,11]. Recent connectomics and resting-state functional MRI studies further indicate abnormal large-scale network connectivity, supporting the view of FM as a systems-level disorder of pain modulation and salience processing [12,13].

In parallel, dysregulation of stress responsive systems has been increasingly recognized as a critical factor in FM. Altered hypothalamic–pituitary–adrenal (HPA) axis functioning, autonomic nervous system imbalance, and neuroimmune interactions contribute to the amplification of pain and to symptom variability [14,15,16]. Stress-related dysregulation manifests not only as altered cortisol rhythms but also as heightened sympathetic tone, reduced parasympathetic activity, and impaired feedback loops. These biological changes have profound behavioral consequences, including fatigue, poor sleep, anxiety, and increased pain sensitivity. These findings align with the conceptualization of FM as a disorder of maladaptive sensory-affective integration at the interface of biological stress systems and central pain processing [8]. Such perspectives challenge earlier notions of FM as a primarily musculoskeletal condition and reinforce the need to investigate neurobiological and psychosocial mechanisms jointly [17].

Despite these advances, diagnosis remains largely symptom based. The 2016 revisions to the American College of Rheumatology (ACR) criteria provided an important framework for standardizing diagnosis, yet they do not fully account for the heterogeneity of FM presentations and lack biological specificity. Similarly, international definitions of nociplastic pain have emphasized central mechanisms but still fall short of offering clinically actionable biomarkers [18]. Consequently, there is an urgent need for innovative diagnostic approaches that can capture the multidimensional complexity of FM and provide objective markers for patient stratification [19,20].

One promising yet underexplored dimension is sensory processing dysfunction. Sensory modulation disorder (SMD) is characterized by impaired regulation of responses to non-noxious sensory input and has been extensively described in neurodevelopmental and psychiatric populations [21]. Its manifestations—sensory hypersensitivity, avoidance behaviors, and maladaptive coping strategies—are strongly associated with elevated stress and diminished adaptive functioning [22]. Recent work indicates that aberrant sensory processing may be relevant to nociplastic pain phenotypes, potentially contributing to both diagnostic uncertainty and symptom persistence [23,24,25]. Despite growing interest in sensory modulation, only a limited number of studies have systematically examined SMD in FM [26]. More recent evidence confirms these observations, showing that fibromyalgia is linked to increased subjective sensory sensitivity across multiple senses [27] and that specific sensory disturbances and avoidance patterns differentiate FM from other pain populations [28]. However, sensory modulation disorder (SMD) has not yet been systematically evaluated in FM using standardized tools such as the Adolescent/Adult Sensory Profile (AASP) [26,28].

Mounting evidence also highlights the interplay between sensory dysregulation and stress physiology in FM. Patients commonly display heightened stress reactivity, atypical cortisol regulation, and autonomic imbalance, which may exacerbate sensory over-responsivity and overall symptom burden [26,29,30]. In addition, recent physiological research has shown altered heart rate variability responses to cognitive stress in FM, underscoring the role of autonomic dysregulation [31]. By jointly assessing sensory modulation and stress mechanisms, it may be possible to delineate diagnostic profiles that reflect both neurophysiological and psychosocial processes. Such multidimensional markers could improve diagnostic timeliness, reduce misclassification, and support personalized treatment stratification [30,31,32]. Consistent with this rationale, neurophysiological investigations have proposed a potential brain-based signature for FM, further underscoring the biological validity of this construct [33]. To our knowledge, this is the first study to systematically evaluate SMD as a diagnostic construct in FM using validated instruments in a relatively large cohort.

Beyond traditional models, recent conceptual advances underscore the relevance of sensory modulation disorder as a bridge between pain and stress physiology. Bar-Shalita and Granovsky [23] proposed that SMD may serve as a mechanism linking nociplastic pain with stress reactivity, offering a unifying framework for understanding overlapping clinical features. Complementary evidence from Groven and colleagues [17] highlights the pervasive role of stressors in shaping FM experiences, suggesting that stress is not simply a consequence but a central driver of symptom expression. Furthermore, Hamam and co-workers [14] showed that dysregulation of the HPA axis and arousal systems in FM may operate synergistically, amplifying sensory abnormalities and clinical symptoms. Together, these findings strengthen the rationale for evaluating SMD not only as a comorbidity but as a potential diagnostic construct in FM, situated at the intersection of sensory and stress pathways [14,23].

Against this background, the present study was designed to: (i) determine the prevalence and characteristics of SMD in FM compared with healthy controls; (ii) examine associations between sensory modulation, perceived stress, and clinical symptom severity; and (iii) evaluate the discriminant validity of sensory profiles for stratifying FM severity. By integrating validated sensory processing and stress assessments, this work aims to clarify the diagnostic significance of SMD and contribute to the development of clinically meaningful biomarkers in FM.

## 2. Materials and Methods

### 2.1. Study Design and Objectives

This cross–sectional, case–control study investigated the diagnostic significance of SMD in FM. The primary objectives were to determine the prevalence and characteristics of SMD among FM patients compared with healthy controls, to evaluate associations between SMD, symptom severity, and perceived stress, and to assess the classification value of sensory modulation profiles for stratifying FM severity.

### 2.2. Participants

A total of 182 adults aged 31–67 years were recruited using purposive convenience sampling rather than consecutive enrollment from a single clinical service. Participants were identified through multiple recruitment channels, including patient associations, rehabilitation centers, rheumatology and pain clinics, social media groups, and community outreach initiatives. This approach aimed to ensure a diverse cohort while reflecting the exploratory, cross-sectional nature of the study. The study population included 104 patients with FM and 78 healthy controls. FM patients were included if they had a confirmed diagnosis of fibromyalgia (FM) established according to the revised American College of Rheumatology (ACR) diagnostic criteria (2010 revision, updated in 2016), which have been in clinical use in Slovenia since 2012. Exclusion criteria comprised neurological or psychiatric disorders, severe systemic illness, or any condition potentially mimicking FM symptoms. Healthy controls were required to be free of chronic pain and psychiatric disorders. All participants provided written informed consent prior to enrollment. Because this was an exploratory, cross-sectional study, the sample size was not determined a priori using formal power calculations. Instead, we aimed to recruit the largest possible number of eligible participants within the study timeframe through patient organizations, rehabilitation centers, social media groups, and clinical practices. The final sample size (*n* = 182) is comparable to or larger than those reported in previous questionnaire-based studies on fibromyalgia, supporting the adequacy of the study’s statistical power for the planned analyses. This sample size was considered adequate for the planned models based on commonly used participant-to-predictor heuristics (≥10–20 participants per predictor). With three primary predictors (sensory sensitivity, sensory avoidance, and perceived stress), the FM subgroup (*n* = 104) provided >30 participants per predictor, thereby ensuring conservative model stability and sufficient statistical power.

### 2.3. Measures

Three validated self-report instruments were used for assessment. The Fibromyalgia Impact Questionnaire (FIQ) evaluates the impact of fibromyalgia on health-related quality of life across ten domains, with total scores ranging from 0 to 100 (higher scores indicate greater disease burden). The instrument showed excellent internal consistency in our sample, with Cronbach’s α = 0.972 at the first measurement and α = 0.929 at the second measurement.

The Perceived Stress Scale (PSS) assesses perceived stress experienced over the previous month using 10 items rated on a 5-point Likert scale. Its test–retest reliability was high (r = 0.869, *p* < 0.001), indicating stable measurement across time points.

The Adolescent/Adult Sensory Profile (AASP) measures sensory processing patterns across four quadrants: low registration, sensation seeking, sensory sensitivity, and sensation avoidance. Test–retest reliability was excellent across all sensory domains (r ≥ 0.859, *p* < 0.001), supporting the stability of the instrument in this population.

All three instruments are widely used and psychometrically validated tools in both clinical and research contexts, and their strong internal consistency and reliability in this study further support their suitability for assessing symptom severity, stress perception, and sensory modulation in individuals with fibromyalgia. Sociodemographic and clinical data, including age, sex, education, comorbidities, and medication use, were also collected via self-report questionnaires. All psychometric and reliability analyses were conducted using IBM SPSS Statistics v29.0 (IBM Corp., Armonk, NY, USA) under the supervision of a professional biostatistician. These included internal consistency analyses (Cronbach’s α) and test–retest reliability assessments based on Pearson correlation coefficients, which confirmed the statistical robustness of all instruments used.

### 2.4. Procedure

All participants completed the AASP, FIQ, and PSS, as well as sociodemographic and clinical questionnaires, at a single baseline assessment. Data were collected anonymously, either online via secure survey software or in paper form using numeric identifiers. No personally identifiable health information was obtained.

### 2.5. Statistical Analysis

Data analyses were conducted using SPSS v29.0 (IBM, Armonk, NY, USA). Descriptive statistics were calculated for demographic and clinical variables. Group differences between FM patients and healthy controls were examined using independent-sample t-tests or chi-square tests, as appropriate. Associations among sensory processing patterns, symptom severity, and stress were evaluated with multiple regression analyses. Discriminant function analysis was employed to assess the classification value of sensory modulation profiles for FM severity. Statistical significance was set at *p* < 0.05. To avoid overfitting, all multivariable analyses were constrained to respect conservative participant-to-predictor ratios. All models remained well within commonly recommended participant-to-predictor thresholds. Before conducting regression analyses, standard assumptions—including linearity, homoscedasticity, normality of residuals, and absence of multicollinearity—were tested and satisfied. To assess the robustness and generalizability of the discriminant model, cross-validation was performed using a leave-one-out procedure, which confirmed the stability of the classification solution. This multi-level analytical approach offered a robust quantitative framework for examining group differences, predictor relationships, and classification performance.

### 2.6. Ethical Considerations

The study was conducted in accordance with the principles of the Declaration of Helsinki. All participants provided informed, voluntary, and anonymous consent to participate in the study. No personally identifiable health information was collected. Given the minimal-risk, questionnaire-based design and the absence of invasive or clinical procedures, formal ethical approval was not required, as the study was classified as minimal-risk behavioral research under institutional policies and national legislation. Data collection and handling were conducted in compliance with the Slovenian Personal Data Protection Act (ZVOP-2) and the General Data Protection Regulation (EU 2016/679).

## 3. Results

### 3.1. Participant Characteristics

A total of 182 individuals participated in the study, comprising 104 patients with fibromyalgia (FM) and 78 healthy controls. The two groups were comparable in age (FM: M = 47.2 ± 8.6; controls: M = 46.1 ± 9.1; *p* = 0.41) and sex distribution (87.5% vs. 83.3% women; *p* = 0.48). Educational attainment and employment status were also broadly similar, indicating that the groups were sociodemographically balanced.

### 3.2. Group Differences in Sensory Modulation, Stress, and FM Symptoms

Baseline comparisons revealed pronounced differences between FM patients and healthy controls (Table 1).

- FM patients exhibited significantly elevated sensory sensitivity and avoidance (both *p* < 0.001), consistent with a sensory modulation disorder profile.
- Clinical outcomes showed a markedly greater disease burden in FM, reflected in higher fibromyalgia impact scores (FIQ; *p* < 0.001) and elevated perceived stress (PSS; *p* < 0.001).
- Additional analyses indicated lower sensation seeking (*p* = 0.002) and slightly higher low registration (*p* = 0.027) in FM compared with controls, further supporting pervasive alterations in sensory processing.

Effect size estimates further supported the robustness of these group differences. Cohen’s d values indicated very large effects for disease impact (FIQ, d = 4.07, 95% CI [3.62, 4.52]), perceived stress (PSS, d = 2.38, 95% CI [2.05, 2.71]), sensory sensitivity (d = 2.14, 95% CI [1.82, 2.47]), and sensory avoidance (d = 1.56, 95% CI [1.21, 1.92]), with medium-to-large effects for low registration (d = 0.54, 95% CI [0.31, 0.77]) and sensation seeking (d = 1.07, 95% CI [0.82, 1.32]).

Taken together, these findings suggest that abnormal sensory modulation co-occurs with increased stress and symptom severity in FM, supporting its consideration as a candidate diagnostic marker with potential utility for early identification and stratification in clinical settings.

### 3.3. Regression Analysis: Stress as a Predictor of FM Impact

To assess the contribution of stress to overall disease impact, a regression model was constructed using PSS scores as the predictor of FIQ outcomes (Table 2).

- Perceived stress was a strong, statistically significant predictor of FM impact (β = 0.57, *p* < 0.001).
- The model explained 31.7% of the variance in FIQ scores (R^2^ = 0.317), indicating that stress substantially contributes to symptom severity and functional impairment in FM.

### 3.4. Discriminant Analysis: Classifying FM Severity from Sensory Profiles and Stress

Discriminant function analysis was performed to evaluate the diagnostic value of sensory modulation patterns and stress in stratifying FM severity (Table 3).

- The discriminant model achieved an overall classification accuracy of 84.1% (Table 3). The function was primarily associated with sensory sensitivity, sensory avoidance, and perceived stress as predictors, with their relative contributions detailed in Table 4.
- Patients with low, medium, and high severity were correctly classified with high accuracy, highlighting the potential of SMD profiles combined with stress measures to support differential diagnosis and patient stratification in FM. However, because the model was derived and tested on the same dataset, these results should be considered exploratory and require replication in independent samples.

The discriminant function analysis produced a statistically significant model (Wilks’ Λ = 0.290, χ^2^ = 219.039, df = 10, *p* < 0.001), indicating strong group separation. The first discriminant function accounted for 98.2% of the explained variance (eigenvalue = 2.304) and showed a high canonical correlation (r = 0.835), underscoring its statistically significant association with disease severity. The second discriminant function was not statistically significant (Wilks’ Λ = 0.959, χ^2^ = 7.493, df = 4, *p* = 0.112).

### 3.5. Standardized Canonical Discriminant Function Coefficients

To further clarify the contribution of individual predictors, standardized canonical discriminant function coefficients were examined (Table 4). The analysis showed that:

- Sensory sensitivity had the strongest loading on the discriminant function, indicating its central role in classifying FM severity.
- Sensory avoidance and perceived stress also contributed substantially, reinforcing their relevance as diagnostic dimensions.
- Together, these predictors accounted for the overall classification accuracy observed in the model (84.1%).

This pattern of predictor contributions highlights the multidimensional nature of sensory and stress-related mechanisms in FM and supports their combined diagnostic value.

## 4. Discussion

### 4.1. Principal Findings

This study provides converging evidence that patients with FM exhibit significantly altered SMD profiles compared with healthy controls. Specifically, FM patients showed heightened sensory sensitivity and avoidance behaviors, accompanied by reduced sensation seeking and mildly increased low registration. These patterns may reflect maladaptive regulation of non-noxious sensory input, extending previous reports of abnormal sensory processing in FM [23]. Importantly, these sensory alterations were strongly associated with greater symptom severity and elevated perceived stress, suggesting that sensory dysregulation may have clinical significance beyond being merely an epiphenomenon. Overall, these convergent abnormalities across sensory domains suggest the presence of a coherent SMD phenotype rather than measurement artifacts.

Regression analyses suggested that perceived stress may be an important predictor of disease impact, explaining nearly one-third of the variance in Fibromyalgia Impact Questionnaire (FIQ) scores. This finding suggests that psychosocial stress may play a central role in shaping disease expression and amplifying functional burden. In parallel, discriminant analysis showed that sensory sensitivity, sensory avoidance, and perceived stress jointly achieved over 80% accuracy in classifying FM severity groups. Such classification performance suggests that the combined assessment of sensory and stress variables could provide substantial diagnostic utility, supporting the conceptualization of SMD as a potential diagnostic construct rather than merely a comorbid feature. Clinically, this means that easily administered sensory and stress measures can add objective structure to severity staging beyond symptom counts alone.

Although these findings are exploratory, observational reports from clinical practice suggest that structured sensory integration approaches may improve sensory responsiveness and stress regulation in patients with FM, which would provide further support for theoretical models of cortical plasticity and stress adaptation [17]. These preliminary observations should be interpreted cautiously, as they are hypothesis-generating rather than confirmatory. Nonetheless, they converge with emerging evidence that sensory-focused strategies can modulate neurocognitive networks implicated in both pain amplification and stress regulation [14]. Taken together, these results suggest that incorporating sensory modulation into FM evaluation provides both mechanistic insight and practical avenues for intervention.

### 4.2. Interpretation in the Context of the Literature

Our findings support the conceptualization of fibromyalgia (FM) as a prototypical nociplastic pain disorder characterized by central nervous system dysregulation [7,10,11,12]. The altered sensory profiles identified in our cohort—particularly heightened sensory sensitivity and avoidance—are consistent with maladaptive modulation patterns previously linked to central sensitization, impaired descending inhibition, and exaggerated stress reactivity [17,18]. In the broader context of pain research, several sensory-related markers have been proposed to characterize nociplastic pain states such as fibromyalgia. These include behavioral indicators, such as heightened sensory sensitivity and avoidance patterns, as well as neurophysiological signatures, including enhanced temporal summation, impaired conditioned pain modulation, and altered cortical network activity observed in functional neuroimaging studies. These results are consistent with neuroimaging evidence showing abnormal connectivity in the insula, anterior cingulate cortex, and prefrontal regions—critical hubs for sensory–affective integration—further supporting the clinical relevance of the sensory markers identified in our study [9,10,11,12,13]. The alignment between behavioral SMD signatures and network-level abnormalities strengthens a mechanistic account in which sensory gain and salience attribution are persistently dysregulated in FM.

A growing body of evidence suggests that nociplastic pain in fibromyalgia arises from dysfunction across a distributed central pain network rather than a single cortical locus. Key regions involved include the insula and anterior cingulate cortex (ACC), which process the affective and interoceptive dimensions of pain; the prefrontal cortex, which contributes to cognitive appraisal and top-down modulation; and the thalamus and primary/secondary somatosensory cortices, which encode sensory–discriminative aspects. Aberrant connectivity among these areas has been consistently observed in functional MRI studies, indicating enhanced central gain and altered salience attribution. Moreover, disrupted activity in descending modulatory pathways—particularly from the periaqueductal gray (PAG) and rostral ventromedial medulla (RVM)—contributes to impaired endogenous pain inhibition. These network-level abnormalities may help explain how non-noxious stimuli can be amplified into persistent pain experiences, providing a neurobiological basis for the sensory hypersensitivity and avoidance behaviors identified in our cohort [9,10,11,12,13].

Crucially, this study contributes to advancing the field. It suggests that sensory dysregulation, when combined with stress measures, may provide high discriminant power for classifying FM severity. Within our discriminant model, sensory sensitivity carried the highest loading on the canonical function, followed by sensory avoidance and perceived stress, and together these predictors achieved >80% classification accuracy (Table 3 and Table 4). This finding suggests that SMD may not merely be an associated feature but could potentially represent a diagnostic dimension with substantial classification value. In practical terms, this pattern supports the use of SMD/stress composites as candidate readouts for future diagnostic algorithms.

These findings are particularly significant given ongoing critiques of the 2016 American College of Rheumatology (ACR) criteria, which remain predominantly symptom-based and insufficiently sensitive to FM heterogeneity [13,15]. It should be noted that all patients in this study were diagnosed according to the revised ACR criteria (2010 revision, with updates introduced in 2016), which have been standard in Slovenian clinical practice since 2012. International pain research initiatives have increasingly highlighted the need for objective markers of nociplastic pain [8,16], yet few studies have rigorously evaluated sensory modulation as a candidate biomarker. Our results therefore begin to address this gap by offering empirical evidence that SMD, in conjunction with stress profiles, enhances diagnostic precision and stratification. Notably, this complements rather than replaces current frameworks, suggesting a tiered approach that pairs standardized symptom criteria with mechanistically informed SMD indices.

In addition, our findings resonate with the broader conceptualization of nociplastic pain as a system-level dysfunction that integrates biological, psychological, and social processes. Unlike structural or inflammatory models, the SMD framework explicitly incorporates sensory gain abnormalities and stress dysregulation, providing a multidimensional lens for understanding FM heterogeneity. This perspective has practical relevance for diagnostics: while neuroimaging studies highlight abnormal connectivity within salience and default mode networks [9,12,13,14], such methods remain resource-intensive and inaccessible in everyday practice. In contrast, standardized sensory assessments capture convergent dysfunctions at the behavioral level and may serve as cost-effective, clinically feasible proxies that reflect mechanisms observed with advanced imaging. This translational link strengthens the case for SMD as a pragmatic biomarker that complements—rather than replaces—more specialized techniques. Consequently, SMD metrics may act as a bridge between cutting-edge mechanistic research and routine clinical decision-making.

The strong association observed between SMD and perceived stress further underscores the bidirectional interplay of neurophysiological and psychosocial processes in FM. Dysregulation of the hypothalamic–pituitary–adrenal (HPA) axis, autonomic imbalance, and neuroimmune alterations have all been shown to exacerbate sensory hypersensitivity and symptom burden [14,15,16,31]. Elevated stress reactivity amplifies pain perception while reducing coping capacity, thereby creating a self-perpetuating cycle of functional impairment [14,29]. Our findings, which link sensory and stress assessments within a unified framework, provide a clinically meaningful lens through which to capture this complex interface more effectively than traditional symptom-based instruments. This also offers a rationale for integrating stress-modulation targets alongside sensory-focused strategies in future multimodal trials.

Although not a primary outcome, independent clinical observations of improved sensory responsiveness and stress regulation with structured sensory integration strategies align with emerging evidence on cortical plasticity and sensory–affective reorganization [14,17,30]. These findings should be interpreted as hypothesis-generating rather than confirmatory, but they highlight the translational potential of sensory-focused approaches to modulate underlying neural networks. Future rigorously designed trials will be essential to establish whether such strategies can meaningfully complement existing FM management paradigms [34,35]. Moreover, a recent scoping review of quantitative sensory testing in fibromyalgia provided additional support for its use as a tool to assess nociplastic pain [36]. Including pre-specified SMD endpoints within such trials would enable direct testing of underlying mechanisms and clinical utility.

Overall, our findings suggest that sensory modulation profiles, particularly when integrated with stress measures, represent a promising diagnostic construct for FM. Beyond supporting mechanistic models of nociplastic pain, these findings provide a framework for developing stratified diagnostic algorithms that move beyond symptom counts toward biologically and psychosocially informed classification. Such algorithms could ultimately shorten time-to-diagnosis and reduce misclassification in complex multimorbid presentations.

### 4.3. Clinical Implications

The high prevalence of SMD among FM patients carries significant diagnostic implications. Incorporating sensory assessments into clinical evaluation could improve diagnostic precision, particularly in distinguishing FM from overlapping conditions such as chronic fatigue syndrome, rheumatoid arthritis, and mood disorders. Unlike many proposed biomarkers, sensory profiles can be assessed using standardized questionnaires that are inexpensive, non-invasive, and easily integrated into routine clinical workflows [29]. This lowers adoption barriers in primary and specialist care and supports scalable implementation.

Beyond differential diagnosis, multidimensional profiling that combines sensory and stress measures offers the potential to stratify FM patients by severity, thereby moving toward more personalized care. Identifying subgroups characterized by pronounced sensory over-responsivity or heightened stress reactivity may allow clinicians to better tailor therapeutic strategies—whether pharmacological, psychological, or sensory-focused interventions. While our exploratory findings tentatively support the utility of sensory-based strategies, robust evidence from randomized controlled trials remains essential before such approaches can be formally incorporated into treatment recommendations [30]. In parallel, while our study used the original FIQ for disease impact, recent work has shown the clinical utility of FIQR severity categories in differentiating outcomes and guiding stratification [37]. Nevertheless, these results underscore the translational potential of sensory assessments not only as diagnostic tools but also as gateways to precision medicine. In addition, embedding SMD screens into routine assessments could facilitate earlier referral, reduce trial-and-error prescribing, and improve patient communication about triggers and pacing.

More broadly, our findings contribute to operationalizing the concept of nociplastic pain. This approach moves beyond symptom counts and offers a pathway toward objective, stratified diagnosis of FM, aligning with international calls for biomarker-driven frameworks in chronic pain research [34]. Linking sensory modulation with stress dysregulation may offer a clinically meaningful perspective on FM heterogeneity and suggests their combined potential as preliminary pragmatic diagnostic biomarkers.

### 4.4. Strengths and Limitations

This study has several notable strengths. It is among the first to systematically evaluate SMD as a diagnostic construct in FM, applying validated instruments with excellent internal consistency (Cronbach’s α > 0.90). The inclusion of both FM patients and healthy controls enabled robust between-group comparisons, while the use of complementary statistical methods—regression for predictive modeling and discriminant analysis for classificatory accuracy—provided a comprehensive evaluation of the clinical relevance of sensory and stress profiles. Moreover, reporting both classification performance and standardized coefficients (Table 3 and Table 4) improves transparency and reproducibility. The relatively large sample size (*n* = 182) further enhances the reliability and generalizability of the findings compared with many previous FM studies, which have often been limited by small cohorts.

At the same time, several limitations warrant careful consideration. The cross-sectional design precludes causal inference regarding the relationship between SMD, stress, and FM severity. The sample was geographically restricted, which may limit the applicability of the results to more diverse populations. Reliance on self-report measures introduces potential response and recall biases, and the absence of objective biomarkers such as neuroimaging, cortisol profiling, or autonomic indices constrains mechanistic interpretation. Finally, the exploratory sensory integration component lacked randomization and sufficient statistical power, and its findings should therefore be regarded as preliminary and hypothesis-generating rather than confirmatory. Future work should also address potential selection biases inherent to purposive sampling and evaluate test–retest stability of SMD measures. Although statistical significance was set at *p* < 0.05, no correction for multiple comparisons was applied. Therefore, the possibility of inflated type I error rates cannot be excluded, and the results should be considered exploratory and interpreted with caution. Furthermore, despite efforts to mitigate overfitting, the discriminant model was both developed and validated on the same dataset, which may have led to overestimation of its classification performance.

Furthermore, our exclusive reliance on self-report questionnaires represents an inherent methodological limitation. Although such instruments are standard tools in fibromyalgia research and clinical practice, future studies integrating clinical, neurophysiological, or imaging biomarkers will be essential to validate and extend our findings.

### 4.5. Future Directions

Future research should prioritize rigorously validating SMD as a diagnostic marker across larger, more diverse populations, ideally including direct comparisons with other chronic pain and stress-related disorders. Integrating sensory assessments with objective physiological and neuroimaging measures could help clarify underlying mechanisms and potentially strengthen biomarker validity [35]. Cross-cohort harmonization of instruments (e.g., AASP variants) and pre-registered analytic pipelines would further enhance comparability.

Longitudinal studies are particularly needed to determine whether SMD profiles predict diagnostic trajectories and treatment responses over time. Such work would establish whether incorporating sensory and stress assessments improves diagnostic timeliness and clinical outcomes. Importantly, future studies should examine whether SMD-based stratification supports precision medicine approaches, enabling interventions tailored to individual neurophysiological and psychosocial profiles. Event-based modeling that links SMD fluctuations with stress physiology and symptom flare-ups could illuminate causal pathways relevant to relapse prevention.

In addition, research should evaluate the stability of SMD profiles across different clinical contexts, including therapeutic interventions and common comorbidities such as chronic fatigue syndrome or mood disorders. Such investigations would clarify whether sensory abnormalities represent trait-like markers or fluctuate with clinical state, which carries direct implications for their diagnostic validity. Combining longitudinal sensory profiling with repeated measures of stress physiology—such as diurnal cortisol rhythms and heart rate variability (HRV)—could reveal dynamic interactions that drive symptom exacerbation and remission [31,38]. Furthermore, incorporating these multimodal markers into machine-learning frameworks may facilitate the development of predictive diagnostic algorithms capable of stratifying patients into biologically meaningful subtypes [39]. Prospective external validation across independent cohorts will be essential before clinical deployment. This precision-oriented direction aligns with international initiatives in personalized pain medicine and underscores the potential of SMD not only as a diagnostic marker but also as a guide for individualized therapeutic targeting. Moreover, the recently proposed grading framework for nociplastic pain offers a harmonized template for operationalizing multimodal markers (including sensory and autonomic readouts) in prospective studies and clinical stratification pipelines [40]. In line with this perspective, the most recent annual review of fibromyalgia research highlighted converging advances in autonomic profiling and small-fiber pathology as adjunct markers that may complement sensory and stress-based assessments [41].

Finally, while our exploratory findings on sensory-focused strategies are preliminary, they provide testable hypotheses for future intervention research. Rigorously designed randomized controlled trials are warranted to evaluate the efficacy of sensory-informed approaches as adjunctive strategies within FM management. Emerging evidence on neuroplasticity and sensory–affective integration provides a strong rationale for this line of inquiry [34,35]. By moving beyond symptom-based frameworks, such research may contribute to establishing clinically meaningful, mechanistically grounded diagnostic markers for FM and may help advance the field toward precision diagnostics and therapeutics. Trial designs that stratify by baseline SMD severity could clarify who benefits most and optimize resource allocation.

## 5. Conclusions

This study suggests that sensory modulation abnormalities are highly prevalent among patients with fibromyalgia (FM) and strongly associated with symptom severity and perceived stress. Multivariate analyses indicates that SMD profiles, in combination with stress measures, showed a preliminary ability to stratify FM severity, pointing to their potential role as diagnostic markers to enhance clinical evaluation and differential diagnosis. Although exploratory, the findings from the sensory integration program further point to the clinical relevance of SMD and suggest avenues for individualized management.

While these results provide novel evidence for SMD as a diagnostic dimension, they should be interpreted in light of this study’s limitations, including reliance on self-report measures and the cross-sectional design. Future studies with larger and more diverse samples, objective biomarkers, and longitudinal follow-ups are needed to establish the robustness and generalizability of these findings. This study successfully achieved its exploratory aims within these methodological constraints, providing initial evidence to guide future research.

Incorporating sensory modulation assessment into FM diagnostics may therefore improve precision, reduce diagnostic delays, and provide a foundation for more targeted treatment approaches, aligning with broader efforts toward precision medicine in chronic pain. By bridging behavioral assessment with mechanistic insights, SMD-based profiling may offer an opportunity to translate advances in nociplastic pain research into scalable clinical practice. Ultimately, such an approach has the potential to reshape diagnostic pathways in FM, enabling earlier recognition, better stratification, and more effective, personalized care.

## Figures and Tables

**Table 1:** Baseline comparisons between FM patients and healthy controls.

Measure	FM Patients (*n* = 104)	Healthy Controls (*n* = 78)	*p*-Value
FIQ	58.1 ± 10.9	18.7 ± 8.2	<0.001
PSS	27.7 ± 4.4	16.3 ± 5.2	<0.001
Sensory sensitivity (AASP)	57.2 ± 7.5	42.5 ± 6.1	<0.001
Sensory avoidance (AASP)	47.1 ± 8.2	35.4 ± 6.8	<0.001
Low registration (AASP)	29.3 ± 4.2	27.1 ± 3.8	0.027
Sensation seeking (AASP)	39.7 ± 5.2	46.2 ± 6.5	0.002

Note: Values are presented as mean ± standard deviation. FIQ: Fibromyalgia Impact Questionnaire; PSS: Perceived Stress Scale; AASP: Adolescent/Adult Sensory Profile.

**Table 2:** Multiple regression analysis predicting fibromyalgia impact (FIQ) from perceived stress (PSS) in FM patients.

Predictor	B	SE (B)	β	t	*p*-Value	R^2^
Constant	27.74	4.43	—	6.27	<0.001	
PSS	1.14	0.16	0.57	6.98	<0.001	0.317

Note: Regression coefficients are based on FM patient data. Model R^2^ = 0.317. SE (B) denotes the standard error of the unstandardized coefficient.

**Table 3:** Classification accuracy of discriminant analysis for FM severity groups based on sensory modulation profiles (AASP) and perceived stress (PSS).

Actual Group	Predicted: Low	Predicted: Medium	Predicted: High
Low (*n* = 98)	84 (85.7%)	14 (14.3%)	0 (0%)
Medium (*n* = 72)	4 (5.6%)	67 (93.1%)	1 (1.4%)
High (*n* = 12)	0 (0%)	10 (83.3%)	2 (16.7%)

Note: Classification based on sensory sensitivity, sensory avoidance, and perceived stress. Overall classification accuracy = 84.1%.

**Table 4:** Standardized coefficients of discriminant function for FM severity classification.

Predictor Variable	Standardized Coefficient
Sensory Sensitivity	0.82
Sensory Avoidance	0.76
Perceived Stress	0.74

Note: Coefficients are standardized canonical discriminant function coefficients. Higher absolute values indicate stronger contribution of the predictor to group discrimination. Overall classification accuracy was 84.1% (see Table 3). Loadings reflect the relative contribution of each predictor to group separation.

## Data Availability

The data supporting the findings of this study are available from the corresponding author upon reasonable request.

## Abbreviations

The following abbreviations are used in this manuscript:

FM	Fibromyalgia
SMD	Sensory Modulation Disorder
CNS	Central Nervous System
PSS	Perceived Stress Scale
FIQ	Fibromyalgia Impact Questionnaire
AASP	Adolescent/Adult Sensory Profile
FIQR	Revised Fibromyalgia Impact Questionnaire
